# You, me, and us: Maintaining self-other distinction enhances coordination, agency, and affect

**DOI:** 10.1016/j.isci.2023.108253

**Published:** 2023-10-28

**Authors:** Merle T. Fairhurst, Ana Tajadura-Jiménez, Peter E. Keller, Ophelia Deroy

**Affiliations:** 1 Centre for Tactile Internet with Human-in-the-Loop (CeTI), Faculty of Electrical and Computer Engineering, Technische Universität Dresden, Dresden, Germany; 2 Munich Centre for Neuroscience, Ludwig Maximilian University, Munich, Germany; 3 i_mBODY Lab, DEI Interactive Systems Group, Department of Computer Science and Engineering, Universidad Carlos III de Madrid, Spain; 4 UCL Interaction Centre (UCLIC), University College London, London, United Kingdom; 5 The MARCS Institute for Brain, Behaviour and Development, Western Sydney University, Sydney, Australia; 6 Center for Music in the Brain, Department of Clinical Medicine, Aarhus University & The Royal Academy of Music Aarhus/Aalborg, Aarhus, Denmark; 7 Faculty of Philosophy, Ludwig Maximilian University, Munich, Germany; 8 Institute of Philosophy, School of Advanced Study, University of London, London, United Kingdom

**Keywords:** Neuroscience, Cognitive neuroscience

## Abstract

Coordinating our actions with others changes how we behave and feel. Here, we provide evidence that interacting with others rests on a balance between self-other integration and segregation. Using a group walking paradigm, participants were instructed to synchronize with a metronome while listening to the sounds of 8 virtual partners. By manipulating the similarity and synchronicity of the partners’ steps to the participant’s own, our novel auditory task disentangles the effects of synchrony and self-other similarity and examines their contribution to both collective and individual awareness. We measured temporal coordination (step timing regularity and synchrony with the metronome), gait patterns, and subjective reports about sense of self and group cohesion. The main findings show that coordination is best when participants hear distinct but synchronous virtual others, leading to greater subjective feelings of agency, strength, dominance, and happiness.

## Highlights

• A group coordination task probed integration and segregation of self and other cues
• More reliable and informative information of self and other facilitates coordination
• Coordination is best when the group provides distinct but synchronous sensory cues
• Greater coordination results in greater feelings of agency, strength, and happiness

## Graphical abstract

**Figure:**
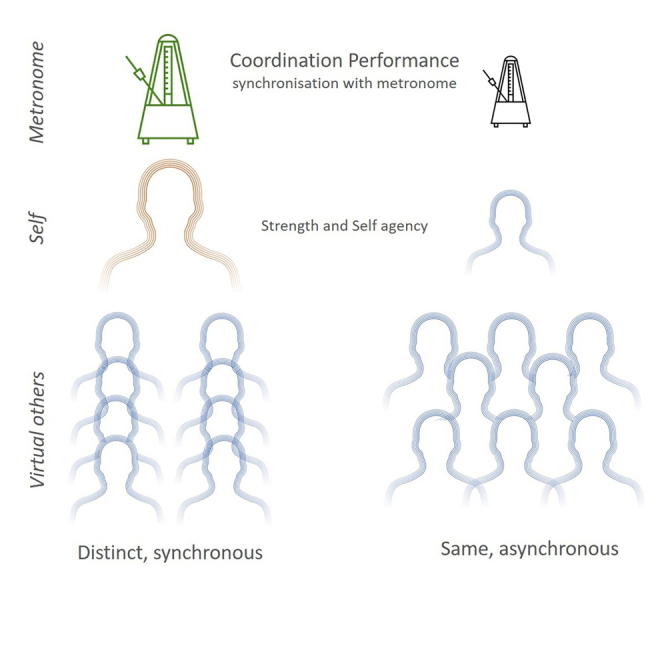

## Introduction

Many social interactions rely on dynamic temporal coordination. In group music making, for example, one player hears another player slowing down or speeding up and changes their tempo accordingly. As such, a significant body of research has used simplified sensorimotor synchronization (SMS) paradigms (e.g., paced finger tapping) and shown that SMS serves as a low-level basis for social cognition.^1^^,^^2^^,^^3^^,^^4^ SMS, which is linked to most forms of joint action, is typically studied by having participants synchronize simple motor behaviors with a sensory pacing stimulus or the sensory feedback of another agent’s actions.^5^ In a musical example, one player hears another player slowing down and changes their tempo accordingly. For the first musician to know if they should change tempo, they need to know which sound was theirs and to compare this to the sound made by the other player. In simplified models of this kind of adaptive behavior (e.g., joint finger tapping), measures can be derived which detail temporal coordination including the degree to which individuals focus on self-tapping (self-focus; standard deviation of inter-step-interval) or the stability of the interaction (self-other focus; standard deviation of asynchronies).^6^ These two measures parallel the suggestion that sensorimotor interaction tasks rely on processing self and other information and a balance between segregation and integration.^2^^,^^7^^,^^8^ Specifically, depending on the reliability and usefulness of the information for optimizing performance, a different combination of self and other information may facilitate coordination.

Coordinating our actions with others may not only change how we *behave* but also how we *feel* and *perceive* ourselves, in particular our own body. From the SMS literature for example, we know that individuals use temporal cues to recognize self-related action (in this case, clapping, see^9^). When walking together with a group of people, we receive sensory bodily information from our own and others’ bodies which is concurrent with our own motor actions. Neuroscience research on mental representations of the body suggest that these are highly plastic and change continuously in response to sensory and sensorimotor bodily inputs,^10^^,^^11^^,^^12^ including sound inputs^13^^,^^14^^,^^15^; for a recent review see.^16^ For instance, the perception of one’s body weight and size can change if the sound of one’s footsteps is modified to suggest a “lighter” body, and these changes are accompanied by feelings of being happier and quicker, as well as by adjustments in gait.^13^^,^^17^ Critically, one of the studies demonstrating such effects also showed that the effect of sound interacted with participants’ body weight, suggesting that both the experimental manipulation and the person’s own body weight need to be taken into consideration when investigating how the sounds we make with our bodies affect one’s body perception and gait.^17^ Do the sounds of a group of people walking with us have similar effects on the mental representation of one’s own body? We regularly hear and integrate the sounds and proprioceptive information of our footsteps along with those sounds of others,^7^ but what if my sounds are more or less similar in timing or quality to the others?

From dyadic accounts, we know that successful interpersonal coordination relies on sensory cues being identified, tracked and processed as relating distinguishably to “self” or “other”. From the social sciences, the suggestion is that a self to other ratio,^18^ that is the degree to which self and other information is processed, is highly variable and will be context dependent.^19^ Previous research suggests that similarity in tempo and sound qualities can affect self-other integration and segregation. Studies of self-recognition highlight the role of temporal information as a potential cue to distinguishing between actions produced by self versus others (see^20^). Across a range of domains, from simple repetitive actions such as clapping^9^ to complex sequential skills such as music performance,^21^ it has been found that people are able to recognize whether sounds in recordings were produced by themselves or others based on temporal cues alone (i.e., when information related to sound quality and intensity is removed prior to the listening test). Individual differences in tempo, onset timing, and the relative duration of sounds thus provide cues to action identity.^20^

It has been shown across various auditory task domains that an individual’s rhythmic movements can unintentionally become entrained to external sequences presented at different tempi. In a basic SMS study that required individual participants to tap a finger in time with a target auditory sequence, Repp (2006) found that the simultaneous presentation of a ‘distractor’ sequence at a slightly (10%) faster or slower tempo attracted taps away from the target.^22^ Even when individuals were able to keep track of the target sequence, the process of resisting attraction to the distractor led to increased timing variability. Such effects of attraction to an external tempo generalize to real interpersonal coordination^23^ even in large groups of skilled performers. A field study of an Afro-Brazilian ritual festival that involves multiple groups of musicians processing independently around town found that some groups deviated from their tempo when within earshot of one another, leading to inter-group entrainment despite the intention to maintain distinct tempi to assert each group’s independence.^24^

**Figure 1:**
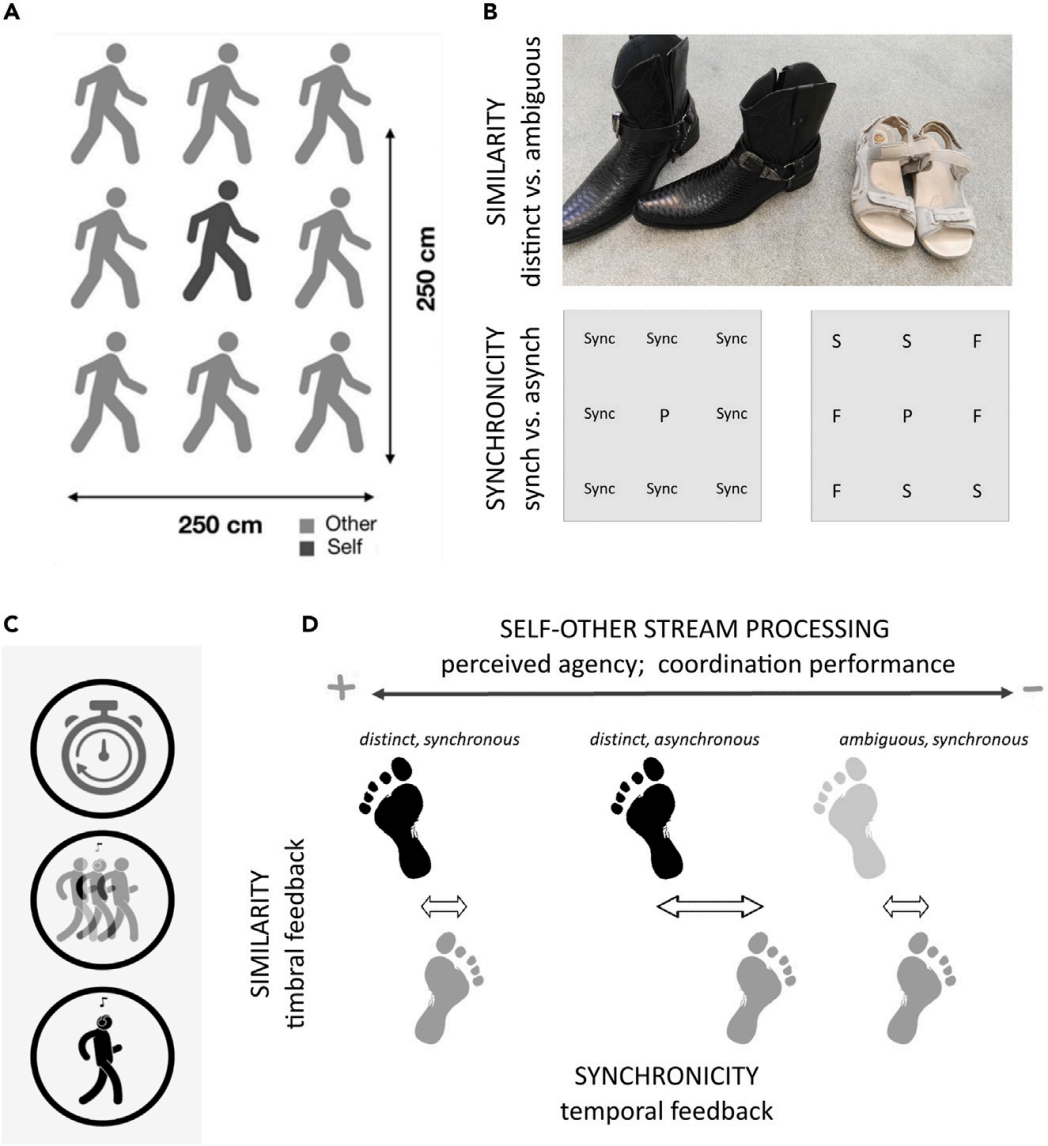
Experiment setup (A) Participants (dark gray) were instructed to walk to the sound of a metronome while listening to the sound of a pre-recorded group of other walkers (light gray), recorded at the following positions relative to the participant. (B) Study design: factors of *similarity* of the sounds made by the “others” relative to “self” (wearing the *same* sandals as worn by the participant or *distinct* cowboy boots), and *synchronicity*, how synchronous the sounds of the group were relative to the metronome to which the participant was instructed to coordinated (synchronous or asynchronous). In the synchronous (sync) condition, virtual partners were recorded walking with the same metronome as the participant (P). In the asynchronous condition, virtual partners were recorded with either a slower (S) or faster (F) metronome than the participant (P). (C) Objective and subjective measures of coordination as measured in terms of temporal coordination, gait differences and subjective reports. (D) Balancing integration and segregation of self and other streams are more likely in the “distinct” conditions when signals of self and other are more easily distinguishable compared to the temporal and timbral overlap in the “same synchronous” condition. In the distinct, synchronous condition, we expect greater perceived agency and greater coordination performance of the instructed task.

Zhang et al. have highlighted the gap between very small (dyad) and very large (crowd^25^) and the need for a more comprehensive understanding of how group size affects coordination and the consequences thereof.^26^^,^^27^ Recently, we investigated coordination dynamics in a group of seven strangers in an ecological environment looking at group synchrony as a function of having a group leader before and after a perturbation.^28^ In this new paradigm, we manipulate the similarity and synchronicity of one’s self with the invisible virtual partners heard to be walking around the participant (Figure 1). The others’ footsteps could be more or less distinct from the quality of the sound made by the participant’s steps. Additionally, these virtual partners are either stepping to the same base metronome beat (synchronous) or the group is made up of individuals walking with either a slower or faster metronome (asynchronous). As a function of these low (SMS) and high (self-attribution similarity) level factors, it is assumed that the information from the virtual partners will be more or less useful for optimizing coordination with the metronome, that is, the explicitly instructed task. When others are in sync, they attract the participant (so this is a second level of coupling, which we don’t directly measure) and effectively boost coupling with the metronome when self-other integration/segregation is optimal (i.e., with distinct sounds). When others are out of sync, they also attract the participant but in this case it can interfere with the primary task (synchronization with the metronome) and weakens coupling (increasing SD asynchrony).

While others have used group walking to explore temporal coordination in groups,^29^ here, we present a combination of both objective and subjective measures of coordination. We use a group walking task to investigate the balance between the integration and segregation of self and other information which can be particularly relevant as group size increases.^30^ As the number of agents interacting increases, the assumption would be that rather than simply either focusing on oneself or the other person (as in the dyad), within a group, one can focus on any one other individual or some subset of the “others”. The integration-segregation problem is thereby more complex and is likely to depend on factors such as predictability, similarity and signal complexity.^31^

Additionally, one may observe changes in how the participant feels about the interaction (competitive vs. cooperative) and also about themselves (agency). Importantly, in the following study, we go beyond the dyad to study an interaction of a medium size group.^26^^,^^28^ Additionally, with this novel paradigm we therefore situate the individual within a group and probe how coordination affects both how an individual moves and feels about their body. The instructed, explicit task was for a participant to walk in synchrony with a metronome (primary, individual goal) while listening to the sounds produced by pre-recorded others. The virtual others were recorded walking in time with the same metronome, to simulate a group where members have the same goal. The design is neutral as to whether this goal happened to be simply the same for each individual, or was represented as common by all. Theoretically, this approach captures the two-folded nature of joint action; that collective intention can only be realized by pursuing an individual action intention as part of the collective act.^32^ We explore the implicit pull of socially-relevant group coordination information and detail how this manifests in terms of measures of self-related focus or integration of self and other information.

Importantly, this differs from previous approaches that measure the causal effects on overall group coordination. Using a group walking task but with virtual auditory partners, we attempt to find balance between non-reductionist approach^33^ and having experimental control. Moreover, we take advantage of the auditory domain which naturally allows for multiple auditory streams which can be listened to individually (segregation) or globally (integration).^34^^,^^35^ Dynamical approaches have described a group of interacting agents as a dynamical system like flashing fireflies and swinging pendulums. A defining characteristic of dynamical systems however is that not only does the system as a unit change across time but the system also shapes individual elements of that system. Here, rather than simply looking for changes in how groups behave, we focus on the effects of group coordination on the individual. Specifically, we predict that coordination performance (step timing regularity and synchrony with the metronome) will be greatest when walking with synchronous others making distinct footstep sounds but also, more importantly, that more synchronous behavior will result in positive effects both on the individual (e.g., strength) and feelings about the group (e.g., greater affiliation). Additionally, based on our previous work, we probe the importance of self-other distinction, maintaining a sense of the “me” in the “we” when coordinating in groups.^36^^,^^37^

## Results

### Temporal coordination while walking with a group of virtual others

**Figure 2:**
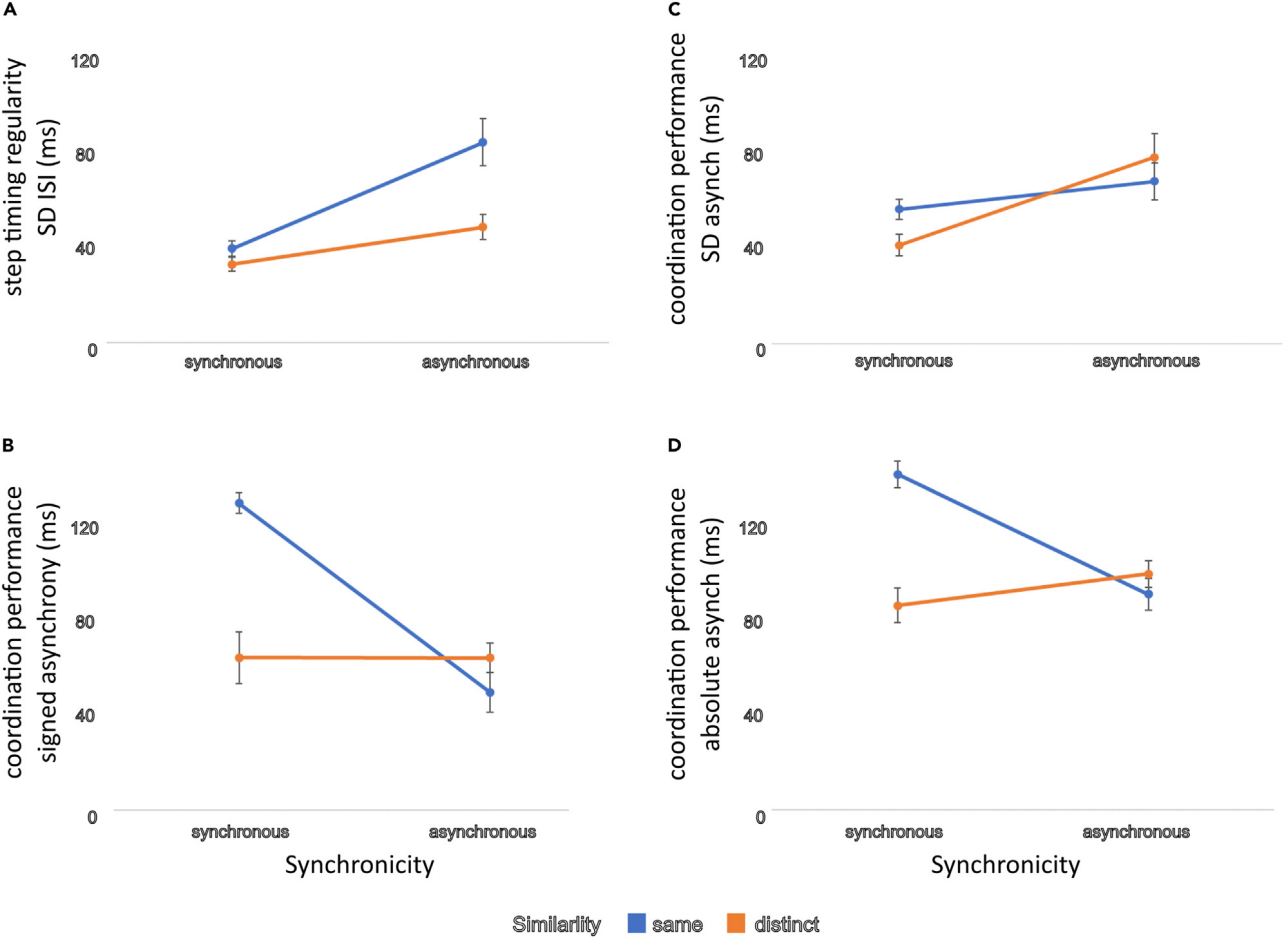
Objective coordination (A–D) Measures of (A) stability of step/action timing (SD interstep interval, SD ISI) and (B) coordination performance (SD asynchrony, where low values indicate good performance) demonstrate significant effects of synchronicity with and similarity of the “others” on individual stepping behavior in time with the auditory metronome (asynchronies are calculated by comparing participant footsteps relative to metronome onsets). Additionally, measures of (C) signed asynchrony and (D) absolute asynchrony show significant main and interaction effects of timbral and temporal similarity. ∗∗ denotes standard error.

To detail temporal coordination at the individual level as a function of the influence of the different auditory input (similarity and synchronicity of virtual others), we used two established measures of SMS. One describes the stability of step/action timing, i.e., step tempo regularity (SD ISI), and the other is a readout of coordination performance (standard deviation of asynchronies, SD asynchrony; synchronization with the metronome with lower values denoting greater synchrony/see Figure 2A). For step timing regularity, we only find a main effect of similarity (*similarity* F(1,24) = 6.842, p = 0.015; *synchronicity* F(1,24) = 3.886, p = 0.06; *similarity ∗ synchronicity* interaction F(1,24) = 1.668, p = 0.209), with lower variability for the distinct conditions. For coordinated performance (SD asynchrony), we find a main effect of synchronicity (F(1,24) = 16.133, p < 0.01) as well as interaction between similarity and synchronicity (F(1,24) = 5.859, p = 0.023) such that performance is best when participants heard distinct but synchronous virtual others (Figure 2B). It should be noted that for this particular task, we see positive asynchronies (rather than the often reported mean negative asynchrony in finger tapping studies) but this should not be surprising given the different form of movement required. As shown in Figures 2C and 2D, the patterns of results for mean signed and absolute asynchronies again show that participants generally produced footsteps that lagged slightly behind the metronome. Repeated measures ANOVAs were run for these additional measures of synchrony with significant interaction and main effects for both signed asynchrony (*similarity*: F(1,24) = 25.80, p < 0.001; *synchronicity*: F(1,24) = 44.56, p < 0.001; *similarity ∗ synchronicity*: F(1,24) = 53.98, p < 0.001, see also Tables S2 and S3) and absolute asynchrony (*similarity*: F(1,24) = 36.08, p < 0.001; *synchronicity*: F(1,24) = 11.32, p < 0.01; *similarity ∗ synchronicity*: F(1,24) = 73.21, p < 0.001, see also Tables S4 and S5).

### Feelings about the self and self-in-other

In order to investigate both the main effects and the interaction between the factors Synchronicity (2 conditions) and Similarity (2 conditions) on the subjective data, we conducted non-parametric analyses of variance (ANOVA) on aligned rank transform data using R software and the ARTool package.^38^ In case of significant interaction effects (i.e., p value <0.05) or trends (i.e., 0.05 < p value <0.1), these were followed by t-tests between conditions, with the corrected p value adjusted with the Tukey method for comparing a family of estimates. Table S1 in Supplementary material shows the Median (Range) for the four conditions for all items as well as the significant effects of the experimental manipulations. In particular, for some items there were significant effects of the factor Synchronicity, and for two items there was a trend toward an interaction of the factors Synchronicity and Similarity.

#### Self and the other

**Figure 3:**
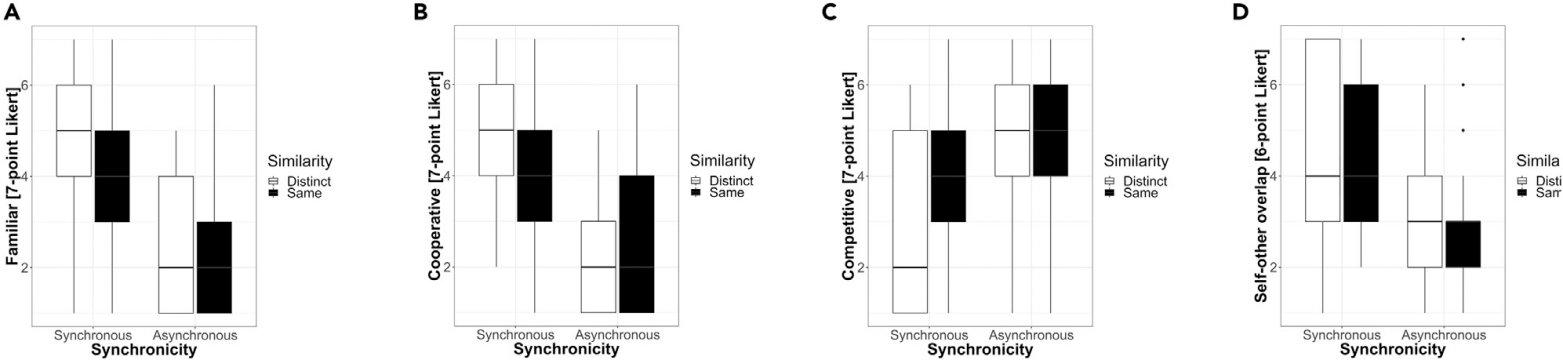
**Self-other feelings**: Questionnaire items used to ask participants whether “the others” seemed (A) Familiar, (B) Cooperative and (C) Competitive. Additionally, participants were asked to rate the degree of (D) self-other overlap. Boxplots showing median and upper and lower hinges corresponding to the first and third quartiles (the 25th and 75th percentiles).

The analyses revealed a significant effect of Synchronicity for all four scales. As shown in Figure 3 (see also Table S1), participants felt that the other were more familiar (F(1,24) = 44.41, p < 0.001), more co-operative (F(1,24) = 56.94, p < 0.001) and less competitive (F(1,24) = 9.20, p = 0.003) in the Synchronous than in the Asynchronous condition. Furthermore, participants reported a higher degree of self-other overlap (F(1,24) = 24.11, p < 0.001) in the Synchronous than in the Asynchronous condition.

There was no effect of Similarity for any of the items and no significant interaction between factors, although there was a tendency toward an interaction between the factors of Synchronicity and Similarity for the items Familiar (F(1,24) = 3.40, p = 0.069) and Competitive (F(1,24) = 3.31, p = 0.073). Follow-up t-tests were all non-significant.

#### Self feelings: Body and emotional feelings

**Figure 4:**
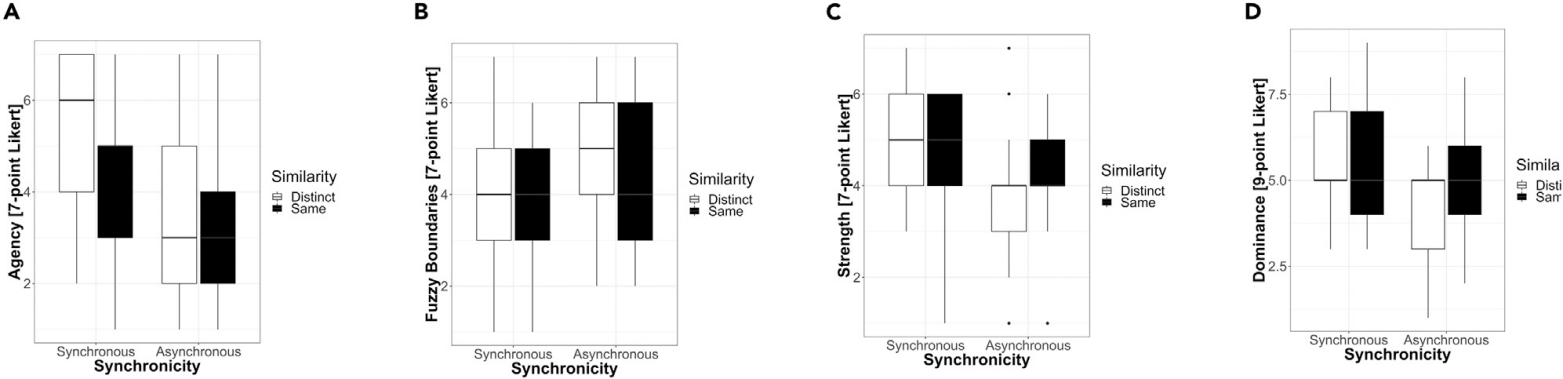
**Feelings about the self**: Questionnaire items related to the self including (A) Agency, (B) Fuzzy boundaries, (C) Strength and (D) Dominance. Boxplots showing median and upper and lower hinges corresponding to the first and third quartiles (the 25th and 75th percentiles).

The analyses revealed a significant effect of Synchronicity for the body feelings scales of Agency, Fuzzy Boundaries, Strength and Straightness, as well as for the emotional feelings scales of Happiness and Dominance. As shown in Figure 4 (see also Table S1), participants felt more agentive reporting that the sounds they heard were produced by their own body (F(1,24) = 27.77, p < 0.001) and that the boundaries of their body were less fuzzy or more vivid (F(1,24) = 5.91, p = 0.017) in the Synchronous than in the Asynchronous condition. Further, they felt stronger (F(1,24) = 9.17, p = 0.003), felt they had a straighter posture (F(1,24) = 8.68, p = 0.004) and felt more dominant (F(1,24) = 11.19, p = 0.001) in the Synchronous than in the Asynchronous condition (see also Figure 3). Finally, participants felt happier (F(1,24) = 10.63, p = 0.002) in the Synchronous than in the Asynchronous condition (see Table S1). There was no effect of Similarity for any of the items and no significant interaction between factors.

### Gait measures and embodied perception

Gait data for 8 participants were lost; we tested our hypotheses with the remaining data from 17 participants. We carried out repeated measures ANOVAs with the factors Synchronicity (2 conditions) and Similarity (2 conditions). We added the participant’s actual weight as covariate as previous studies have shown that the effects of heard footstep sounds on perceived body weight is modulated by actual body weight and that this may variably affect self-related information in some conditions more than in others.^17^ Across a number of variables typically used to probe gait patterns,^17^^,^^39^^,^^40^^,^^41^^,^^42^ we found significant effects for heel exerted force against the ground (i.e., related to applied strength^43^) and stance time. Previous works have related these parameters to actual or perceived body heaviness (e.g.,^17^^,^^41^^,^^42^^,^^43^^,^^44^).

The ANOVA using **heel force** data showed a significant effect of Synchronicity (F(1,15) = 4.99, p = 0.041), and interaction of Synchronicity with body weight (F(1,15) = 5.19, p = 0.038). In particular, the exerted force was bigger in the synchronous (M = 674.21, SD = 29.02; values refer to voltage analog readings) than in the asynchronous condition (M = 673.65, SD = 28.63); the interaction effect reflected that this difference was larger for people with larger body weight. This effect in heel force is consistent with walking with “stronger” and “heavier” steps^43^ in the self-other synchronous (vs. asynchronous) condition. Indeed, in the questionnaire data participants report feeling stronger and more dominant in the synchronous conditions relative to the asynchronous ones (see Figure 4).

In particular, the ANOVA on the **stance time** data showed a significant effect of Similarity (F(1,15) = 4.71, p = 0.047), and an interaction of Similarity with body weight (F(1,15) = 6.50, p = 0.022). In particular, the stance time was bigger in the distinct (M = 1.032 s, SD = 0.09) than in the similar condition (M = 0.86 s, SD = 0.1); the interaction effect reflected that this difference was larger for people with greater body weight. This effect in stance time is consistent with walking with “heavier” steps^41^^,^^44^ in the condition in which the self footstep sounds were distinct (vs. similar) from the sounds produced by the others.

## Discussion

With a novel group walking task, we looked at how coordinating within a group changes how the individual moves and feels when interacting with others. Specifically, we tested participants walking in time with a metronome together with an array of 8 virtual (non-adaptive) auditory partners, and measured changes in physical coordination (step timing regularity and synchrony with the metronome) and gait as well as feelings about the self and group. In so doing, we provide evidence for a mechanism of variable self-other integration or segregation which potentially explains coordination, individual movement and subjective reports. Replicating previous work (see^45^ for a review), in the condition in which self- and other-information overlaps **temporally** (main effect of synchronicity), individuals feel more part of the group (“me in the we”) as well as more familiar, more co-operative and less competitive. Interestingly, it is also in the synchronous conditions that individuals feel the greatest sense of self-agency, complementing previous accounts suggesting the need to maintain the “me” despite being part of the group.^36^^,^^37^

Our study departs from previous studies in several respects, and firstly in the **nature of the task** used. Specifically, our approach provides insight into how feelings about self and others vary when interacting in a medium sized group. Previous research into group dynamics has primarily looked at either smaller or larger groups interacting, with only a handful looking at medium sized groups. Moreover, other studies looking at marching or walking (e.g.,^46^) generally served to investigate overall group synchrony. In these studies, the instructed task typically is to synchronize with the group as a whole. By contrast, in our paradigm, we ask participants to couple with the metronome while measuring the influence of different types of virtual groups walking around the participant (see^47^ for a related study looking at the effect on motion and gaze behaviors as a function of social factors). These different groups of virtual others produced footstep sounds that are either temporally or timbrally different from those of the participant. This enables us to look at the pull of the crowd, testing the hypothesis that the sound of synchronous but distinct “other” footsteps promotes optimal integration and performance.

Within the musical context there are various examples of studies that investigate synchronization dynamics both in dyads and groups. To our knowledge, however, there are no other reports of a group coordination task in which **the effects of auditory information alone on coordination are examined** (the sounds of the virtual partners’ footsteps; for related work in dyads, see^48^^,^^49^). One notable exception might be the “blind” condition in a study investigating head motion and gaze changes in string quartets.^47^ The other is the work by Honisch et al. investigating different synchronization strategies as a function of the richness of sensory cues.^50^ In the present study, we focus on auditory information as various studies highlight its importance in providing potent cues for group synchrony by inducing a regular beat that compels one to move,^5^^,^^51^^,^^52^^,^^53^ while not relying on a specific direction of gaze (which can be constrained when in large groups on the move). Moreover, by using pre-recorded real human walking sounds, this design provides both ecological auditory cues with additional experimental control in that visual (superficial “like-me”) features that may dictate in-group/out-group classification which might influence information processing are not available.

The present study further differentiates itself with the **kinds of measures used to describe how the participant felt and behaved in the various social contexts**. Past research looking at medium-sized groups has been either more observational in nature^16^ or has focused on describing the sensorimotor dynamics between interacting individuals.^26^^,^^29^^,^^46^^,^^54^^,^^55^^,^^56^^,^^57^^,^^58^^,^^59^ This research has as its aim to objectively quantify the degree of coupling between interacting individuals. One of the first of these by Richardson et al.^60^ studied groups of six naive students in rocking chairs, while a more recent example is a study by Alderisio et al.,^61^ where they manipulated information exchange to measure topological changes between two groups of seven individuals waving arms. In the present study, we take the first step into observing differences at three levels of coordination-related changes as well as identify future targets. Our results suggest that temporal and timbral qualities of the sounds we make when we walk together change the way we walk and feel about being in a group. Specifically, measures of these changes vary as a function of the similarity (i.e., timbral overlap or how similar the sound of the group of others was to the participant) and synchronicity (i.e., temporal overlap or how synchronized the others’ footstep sounds were with the participants, by virtue of alignment with the metronome).

Exploring how participants fared at the instructed task (coordinating with the metronome), the SD asynchrony measure shows an interaction of similarity and synchronicity such that performance was best when participants heard distinct but synchronous virtual others. To perform the explicit task of synchronizing with the auditory metronome, we assume that the participant benefits from integrating self and other related information in the synchronous condition. We posit that this is easiest when there is distinct, i.e., timbrally different, information but which is temporally closer in time. The effect of temporal overlap builds on existing work in the music domain which has demonstrated that differences in action timing, in this case ambiguity in the onset of the virtual partners onset and that of the participant in the synchronous condition, can influence the quality of interpersonal coordination and synchronization. Pianists are better able to synchronize with recordings of their own performances than others’ performances of complementary parts in virtual duos even after delays lasting several months.^62^ Moreover, synchrony in real piano duos is high when members of the duo have similar preferences about overall tempo^63^ and similar goals concerning local tempo changes.^8^^,^^64^ However, the match presumably does not need to be perfect for self-other integration to occur, given that humans are flexibly able to interact with a range of partners.

Whether the degree of temporal influence by others is affected by sound similarity is an open question. As with similarity of visual information, similarity in terms of acoustic properties might strengthen entrainment to others to the extent that it encourages self-other integration. Although auditory distractor effects observed by Repp were not affected by pitch distance (2 vs. 20 semitones), if sounds are identical across sequences, attraction might be especially compelling due to perceptual integration of sequences into a single auditory stream.^22^ Related effects were found in a study of simultaneous intra-personal and inter-agent coordination that required participants to tap with alternating hands in antiphase with an external metronome.^65^ Performance was poorer when taps by each hand triggered tones that were identical to the metronome tones than when taps triggered tones that were distinctive but close in pitch to the metronome. In our paradigm, to fulfill the instructed task of synchronizing with the metronome in the asynchronous condition, the participant presumably has to be able to detect her or his own footsteps reliably and resist entrainment to the asynchronous virtual others. Both processes (detecting self and resisting entrainment to others) presumably benefit from self-other segregation, which might be more difficult if the footstep sounds are timbrally similar than when they are distinct with similar sounds favoring integration into a single auditory stream.

It is likewise an open question whether sound similarity would affect synchronization with the metronome when virtual partners are walking at the same tempo. In this case, sound similarity could facilitate coordination by strengthening entrainment (assuming that the virtual partners are in precise synchrony with the metronome). However, reliably detecting one’s own footsteps might be compromised to some degree by auditory masking by others’ footstep sounds (though tactile and proprioceptive feedback are still available). A study of choir singing found that interpersonal synchrony in physiological measures (respiration and heart rate variability) was higher when singing in unison than when singing with multiple voice parts.^66^ However, research focusing on coordination of the sounds themselves has yielded little evidence for benefits of pitch-based similarity. The magnitude of asynchronies in studies of piano and vocal duos was not affected by whether paired individuals produced the same pitches (unison) or different pitches (octaves or rounds, where pitches in one part lag behind the other).^63^^,^^67^^,^^68^ Participants in these studies were experienced ensemble musicians, which leaves open the possibility that producing the same sounds may be disruptive in less experienced individuals. Specifically, ambiguity of agency due to impaired ability to distinguish between sounds produced by self and others might interfere with the process of making appropriate adjustments to correct for asynchronies.^36^^,^^37^

For the stability of action timing (step tempo), we see a significant main effect of similarity and an approaching significant effect of synchronicity. Step timing was less variable when the footsteps of self and other were distinctive. Previous work on self-paced movement (i.e., without an external auditory pacing sequence) indicates that whether the task is performed using closed-loop (relying on feedback) or open-loop (not relying on feedback) control can vary depending on the task demands and the strategy employed by the individual.^69^^,^^70^ The present results suggest closed-loop control where the participant is best able to walk at a stable tempo when receiving unambiguous feedback about the timing of their own actions. The marginal effect of synchronicity may be a case of period locking due to entrainment (which is perhaps captured more directly by the SD of asynchronies measure), which produces timing adjustments that introduce systematic variability that normally serves to reduce global asynchrony.^65^

Turning to the subjective experience of walking within a group, we find a main effect of synchronicity on subjective reports relating to the experience of self relative to others with significantly higher ratings of cooperativeness and self-other overlap but with less fuzzy boundaries in the conditions of greater temporal overlap. This corroborates both anecdotal experience^71^ and empirical data^36^^,^^72^ that suggests that doing things with others in time leads to greater feelings of oneness. Perhaps more striking is that when we consider how doing things with others changes how we feel about ourselves (i.e., at the individual/“me” level), we find that it is in the synchronous conditions when individuals report feeling the greatest sense of self-agency. This provides a nuance to the prevailing idea that coordinating in groups (even simply imagining walking in a crowd) leads to deindividuation.^58^ Instead, our findings highlight the need to maintain the “me” despite being part of the group.^36^ Indeed, participants also felt that the boundaries of their body were less fuzzy or more vivid in the synchronous conditions when their behavior matched that of synchronous virtual others.

Further, our results also confirmed the effects of group coordination on self-body perception, as participants reported feeling stronger and with a straighter posture in the synchronous conditions, in which participants also felt happier and more dominant than in the asynchronous conditions. These reports of feeling stronger and more dominant when coordinating with others are consistent with the effects observed in gait in terms of higher applied heel force^17^^,^^43^ in the self-other synchronous (vs. asynchronous) condition, showing an agreement between implicit and explicit measures of self-body perception. Linking the two levels of observation, the effect seen on heel strikes in the synchronous condition can be related to previous work of SMS^73^ from which one could infer that increased feel force could be due to participants attempting to generate more feedback about their own steps in order to disambiguate their action outputs from those of the others but increasing information from complementary proprioceptive and tactile channels. These effects on self-body perception are also consistent with previous reports showing that synchronous multisensory or sensorimotor signals between self and other can change self-body perception. For instance, seeing another person’s body being touched while feeling touch on one’s own body (e.g.,^13^^,^^74^), or seeing another person moving in the same way and at the same time that one’s body is moving^75^^,^^76^ can result in the assimilation of features of the other’s body in the mental representation of one’s own body and in finding self-other equivalences which form the basis of social behavior^77^^,^^78^“like “me” processes^77^^,^^78^). The positive emotional feelings that derived from the synchronization with others in this study further suggest positive consequences of feeling part of a group. Importantly, the gait data also showed an effect of similarity of the self-other sounds on how people walked, with participants spending more time in contact with the floor in the condition in which the self footstep sounds were distinct (vs. similar) from the sounds produced by the others, an effect consistent with walking with “lighter” steps^41^^,^^44^ when sounding the same as others.

Considering the objective measures of coordination and gait alongside subjective judgments of feelings and states is potentially informative about different levels of social-cognitive processing. Observing effects of synchronicity at both objective and subjective levels, but reliable effects of similarity only on objective measures, suggests a hierarchy of social cues. Specifically, temporal cues about the contingency and coupling of actions between group members may influence interpersonal alignment across behavioral and experiential levels, while the influence of similarity in acoustic properties is restricted to behavioral alignment. This apparent dissociation in the effects of timing and spectral cues may reflect the primacy of information about onset timing in determining and signaling the quality of interpersonal coordination.^22^^,^^79^

### Conclusion

Coordinating in groups by dancing or marching has been suggested to serve as a form of “muscular bonding”^71^ and to increase group affiliation and feelings of connectedness.^80^^,^^81^ Other effects may however also show at the individual level, for instance as a form of personal enlargement.^71^ By manipulating how similar and synchronous the group is in which an individual interacts, we investigate contexts in which individuals variably integrate or segregate self and other information. This is done by comparing differences at three levels of observation, namely gait, synchronization behavior and subjective ratings. Main findings indicate that temporal coordination benefits from distinct but synchronous virtual others, leading to increased subjective feelings of agency, strength, dominance, and happiness. We refocus the discussion of group dynamics on the individual (the “me” in the “we”) by not only measuring group affiliation and cohesion but rather also describing how the person moved and felt about their body.

### Limitations of the study

Our results suggest that the way we walk together changes the sound we produce, and the sounds we make together changes the way we walk and feel about being in a group. The causality of this relationship between these levels of observation needs to be investigated in the future. Additionally, the current study was limited by not capturing the sensorimotor coupling between the individual and the group of others (only the synchronization with the metronome), mainly due to multiple potential synchronization targets among the group. In future studies, we plan to investigate the different ways of measuring this second level of coupling and testing whether this is done by coupling to the average onset of the group, the leader in the group or the participant’s nearest neighbor. Another alternative would be to vary properties of synchrony within the group parametrically. It would also be of interest to investigate more dynamic changes in coupling across an individual trial rather than simply capturing an average. Lastly, the study of group behavior and dynamics would profit from determining the nature of the scaling effect to determine whether the balance of self-other integration and segregation varies in a reliable manner as a function of group size. Addressing these outstanding issues would contribute further to our understanding of psychological mechanisms that regulate self-other relations in real-time coordination, and their consequences for sense of self and social connectedness. Finally, although recruited participants were approximately balanced in terms of sex and socioeconomic status, factors of sex, gender, ancestry, race, and ethnicity were not collected nor were these factors or a combination of these factors analyzed. As a result, it should be noted that this potentially limits the study’s generalization. Future studies would benefit from exploring how these important individual differences (and the make-up of a particular group as a function of the factors) affect how groups interact.

## STAR★Methods

### Resource availability

#### Lead contact

Further information and requests for resources and reagents should be directed to and will be fulfilled by the lead contact, Merle Fairhurst (merle.fairhurst@gmail.com).

#### Materials availability

This study did not generate new materials.

#### Data and code availability

All coordination, gait and subjective report data can be found on Mendeley Data (Fairhurst, Merle (2023), “Group walking”, Mendeley Data, V1: https://doi.org/10.17632/4kkfffmtk5.1) and are publicly available as of the date of publication. This paper does not report original code. Any additional information required to reanalyze the data reported in this paper is available from the lead contact upon request.

#### Study participant details

The experiment was conducted at the Center for the Study of the Senses, University of London. With both verbal and written instructions, 25 Participants (16 female, M_age_ = 26.8(±7.8)) were briefed as to the broad nature of the study. Sample size for this study was determined based on previous experiments in human temporal coordination^6^^,^^82^ and an a prior G∗Power analysis.^83^ For our 2 × 2, repeated measures within subject design, testing for a medium effect size (d = 0.25), and an alpha of 0.05, the result showed that a total sample of 24 participants was required to achieve a power of 0.80. Ethics approval for the experiment was obtained from the School of Advanced Study, Research Ethics Committee. Written informed consent was obtained from all participants and the experiment was performed in accordance with the relevant guidelines and regulations of the School of Advanced Study, University of London.

### Method details

#### Study design

This group walking paradigm requires participants to walk on the spot and in synchrony with a metronome while listening to the sounds of other individuals walking around them. The auditory metronome had a set inter-stimulus interval (ISI) of 500 ms, which corresponds to a comfortable rate (2 Hz) of human locomotion.^84^ The participant wore a pair of headphones while blindfolded and was told that they would hear the metronome as well as the sounds of their own footsteps and those of others walking around them. The sounds of those “others” or “virtual partners” (VPs) were in fact the pre-recorded sounds of eight individuals’ footsteps as they walked in time with a set metronome at either 500 ms (synchronous condition) or 425/575 ms (asynchronous condition); a first factor of ***synchronicity***. The asynchronous condition was created by compiling an audio file in which half of the virtual partners in the group were walking at the slower tempo (575 ms) while the other half walked at the faster tempo (425 ms). The virtual partners were also recorded walking in either the same footwear, sandals (“same”), or different footwear, boots (“distinct”) as the participant; two levels of a second factor of ***similarity***. Six “asynchronous” stimuli were created for both the **distinct** and **same** conditions. These were created by randomly selecting either the faster or slower recorded session for each of the 8 confederates so that there were always half of the confederates leading and half following. Two sound files were created by combining these auditory streams with two configurations of slow and fast confederates and, from these, three sound samples were extracted. Similarly, there were three **synchronous** stimuli each created for the **distinct** and **same** condition. All stimuli were 23 s in length made up of 2 s of silence, followed by two metronome beats (no sound of footsteps) followed by the sound of footsteps with metronome for approximately 20.5 s. For each participant, a random selection of these stimuli was selected and presented. In this 2 × 2 repeated measures design, we probed an interaction between synchronicity and similarity with our data, testing the hypothesis that coordination (step timing regularity and synchrony with the metronome) is optimal when listening to distinct but synchronous virtual others.

#### Apparatus and materials

Recordings were done in a quiet room with dimensions 490 × 645 × 280 cm (length, width, height) with carpet flooring. A 250 × 250 cm square with a 3 × 3 matrix of walker positions was created in the center of the room, hereinafter referred to as “self-other matrix” (see Figure 1). The participant (“self”) is to be placed at the center of the matrix, while eight “other” walkers are placed at the center of the remaining eight positions. Each walker position is defined by a 50 × 50 cm square. Each square is separated by 50 cm from the others. A wooden board with dimensions 44.5 × 89 cm was placed at each walker position for walkers to produce clear, distinctive sounds when walking on it. A pair of binaural microphones (Core Sound, frequency response 20 Hz-20 kHz) that attached to a person’s ears, a sound card (RME FirefaceUC) and a laptop computer were used to record the sound stimuli (the footstep sounds of the “others”) used in the experiment. The microphones were worn by a person standing at the center of the self-other matrix (i.e., at the “self” position). Further, a piezo sensor (Schaller Oyster723) was placed on the floor to record the footstep impulses.

During the experiment, two microphones (Core Sound) were attached to the participants’ sandals to capture their walking sounds. A sound card (RME FirefaceUCT) and Max/MSP software was used to mix the microphone inputs with the pre-recorded sounds of others and the metronome signal. The resulting sound was fed back via closed headphones (Sennheiser HDA300) with high passive ambient noise attenuation (>30 dBA) that muffle the actual sound of footsteps. In addition, the software recorded the self-other sounds, together with the input from the piezo sensor (Schaller Oyster723) placed on the floor near the participant, for post-analysis. Piezo recordings of the participant and pre-recorded “others” were used to extract onset data, used to quantify objective coordination. Piezo sound files were analyzed for peaks (i.e., contact with the floor) using a MATLAB script. Participant onsets were then used to calculate mean and standard deviation of the asynchronies between the participant footsteps and metronome. The part of the system dedicated to measure gait data is comprised of four force sensitive resistors (FSR; 1.75 × 1.5″ sensing area) attached to the front and the rear part of the sandal insoles; and two 9-axis MotionTracking devices (MEMS; Sparkfun MPU-9150) which are placed on the participant’s ankles. FSRs and MEMS in each foot connect to a Microduino microcontroller board, which combines a Microduino Core, a Microduino Shield Bluetooth 4.0, and a Microduino USBTTL Shield, and a battery. This board is placed into a plastic box and attached to the sandals, linking the sensors via Bluetooth to a smartphone. An especially developed app (SmartShoes) captures for each foot, 3-axis acceleration, 3-axis gyroscope and FSR data and saves it as TXT file on quitting.^17^

#### Measures

##### Subject reports

After each experimental block, participants sat down and were asked to fill in four questionnaires. Firstly, a questionnaire assessing **self-other feelings**. This questionnaire included three 7-point Likert-type response items asking about whether “the others” seemed familiar (item ranging from “not familiar to me” to “very familiar to me”), cooperative (item ranging from “not cooperative at all” to “highly cooperative”) and competitive (item ranging from “not competitive at all” to “highly competitive”). In addition, to these items, we used the inclusion of the other in the self scale^85^; 7-level Likert-type response item^86^) to quantify feelings of affiliation with the other walkers (self-other overlap).

Secondly, a questionnaire consisting of 7-point Likert-type response items was used to quantify the participants’ **body feelings** (based on the questionnaire used in^17^^,^^41^^,^^42^^,^^87^). Participants were asked to rate their level of agreement with three statements (ranging from strongly agree to strongly disagree) regarding whether they felt the walking sounds they heard were produced by their own footsteps/body (agency), that their body boundaries were less vivid than normal (fuzzy boundaries) and that they could tell where their feet were (feet localization). Four additional 7-point Likert-type response items were used to quantify other body feelings. These items ranged from “slow” to “quick” (body speed), “light” to “heavy” (body weight), “weak” to “strong” (body strength) and “crouched, stooped” to “elongated, extended” (body straightness). Finally, the **emotional state** of participants was quantified by using the valence, arousal and dominance 9-item graphic scales of the self-assessment manikin questionnaire.^88^

##### Gait data

Raw FSR and acceleration sensor data were analyzed to extract gait biomechanics related to implicit changes in perceived body weight.^17^^,^^41^ FSR data served to quantify the exerted force of heel and toe against the ground and their contact times, as well as the stance and the gait cycle times. To measure force, we recorded the voltage response (analog readings from 0 to 1024), which exhibits a linear relationship with the force applied against the ground. As the force applied against the ground increases, the resistance of the FSR decreases, and the voltage is directly proportional to the inverse of the FSR resistance. MEMS data quantified the foot lifting/downward acceleration during the swing phase.^39^^,^^89^ A specifically developed piece of software (described in detail in,^87^ was used to extract the gait parameters. Data from the left foot were extracted and used for the analysis (as in^41^), with the rationale that this was synchronised with the upbeat. The net acceleration was calculated as the square root of the sum of the squares of the 3 axes (Tajadura-Jimenez et al., 2015); data were low-pass-filtered to reduce noise,^90^^,^^91^ and the first derivative was calculated. For FSR data, the foot is considered to touch the ground when the FSR value exceeds a threshold value; erroneous detections of the foot leaving the ground are avoided by considering the rate of acceleration change. Once all steps had been identified within the datasets, we extracted for each foot and step these parameters: mean exerted force of heel/toes against the ground, stance or contact time (difference between initial strike time and last contact time), gait cycle time and foot upward/downward acceleration. For each trial and parameter, we calculated the average of all steps in the walking phase.

##### Temporal coordination data

As mentioned previously, participant onset data was recorded and used to describe performance on the instructed task, namely coordination with the metronome as a function of the sounds of others. Here we report classical temporal coordination measures typical to other joint action (sensorimotor synchronisation) tasks. The first of these is the standard deviation (SD) asynchrony, a measure of interaction performance or coupling between two time series.^65^^,^^82^ This measure is obtained by calculating the asynchronies across a trial between the participant and the auditory metronome and calculating the standard deviation per trial, per condition per participant i.e., probing coordination performance of the instructed synchronisation task. Lower SD asynchrony is indicative of greater coupling and assumedly an optimal balance of self/other integration and segregation. The second measure reported here is that of the SD ISI, a measure of step timing regularity, or tempo stability. From previous work, it is assumed that a lower SD ISI indicates that the agent is focusing on producing a reliable signal.^6^^,^^65^ For each participant, the timecourse for each trial (by condition) can be analyzed by calculating each inter-step-interval and then calculating the standard deviation. Step timing regularity is a necessary but not sufficient condition for coordination performance. Moreover, while this measure of action timing stability describes how an individual (within a group) focuses primarily on self-related information (segregation), a measure of SD asynchrony highlights the effect of our manipulation on performance of the set coordination task.

## Supplemental information

Document S1. Tables S1‒S5
