# Supplementary material for: You, me, and us: Maintaining self-other distinction enhances coordination, agency, and affect
Source: iScience. 2023 Oct 28;26(12):108253. doi: 10.1016/j.isci.2023.108253 (PMC10679890; doi:10.1016/j.isci.2023.108253)
Supplement: Document S1. Tables S1‒S5 [file mmc1.pdf]

iScience, Volume *26*

Supplemental information

You, me, and us: Maintaining self-other distinction enhances coordination, agency, and affect

Merle T. Fairhurst, Ana Tajadura-Jiménez, Peter E. Keller, and Ophelia Deroy

Supplementary Material

You, me and us: Maintaining self-other distinction enhances coordination, agency and affect

[1]Centre for Tactile Internet with Human-in-the-Loop (CeTI), Faculty of Electrical and Computer Engineering, Technische Universität Dresden,

[2]Munich Centre for Neuroscience, Ludwig Maximilian University,

[3]i_mBODY lab, DEI Interactive Systems Group, Department of Computer Science and Engineering, Universidad Carlos III de Madrid,

[4]UCL Interaction Centre (UCLIC), University College London,

[5]The MARCS Institute for Brain, Behaviour and Development, Western Sydney University,

[6]Center for Music in the Brain, Department of Clinical Medicine, Aarhus University & The Royal Academy of Music Aarhus/Aalborg

[7]Faculty of Philosophy, Ludwig Maximilian University,

[8]Institute of Philosophy, School of Advanced Study, University of London

Table S1. Median(Range) for questionnaire data (7-level Likert items except for the 9-level valence, arousal and dominance scales) for the whole group of participants and the four conditions. Related to Figure 3 and Figure 4. P-values for significant effects and trends are indicated. * marks significant differences due to Synchronicity and ^ marks a trend towards an interaction of the factors Synchronicity and Similarity.

Measures	Synchronous		Asynchronous		Significant effects (p-values)
	Distinct	Same	Distinct	Same	
Familiar	5 (1-7)	4 (1-7)	2 (1-5)	2 (1-6)	*(p<0.001)<br>^(p=0.069)
Cooperative	5 (2-7)	4 (1-7)	2 (1-5)	2 (1-6)	*(p<0.001)
Competitive	2 (1-6)	4 1-7)	5 (1-7)	5 (1-7)	*(p=0.003)<br>^(p=0.073)
Self-other overlap	4 (1-7)	4 (2-7)	3 (1-6)	3 (1-7)	*(p<0.001)
Agency	6 (2-7)	5 (1-7)	3 (1-7)	3 (1-7)	*(p<0.001)
Fuzzy Boundaries	4 (1-7)	4 (1-6)	5 (2-7)	4 (2-7)	*(p=0.017)
Speed	5 (1-7)	4 (1-6)	4 (1-7)	4 (1-6)	n.s.
Weight	4 (1-7)	4 (1-7)	4 (2-7)	4 (2-7)	n.s.
Strength	5 (3-7)	5 (1-6)	4 (1-7)	4 (1-6)	*(p=0.003)
Straightness	5 (1-6)	4 (1-7)	4 (1-6)	4 (1-6)	*(p=0.004)
Feet localization	5 (2-7)	5 (2-7)	4 (1-7)	5 (1-7)	n.s.
Valence (Happiness)	7 (4-9)	6 (3-9)	5 (1-8)	5 (2-8)	*(p=0.002)
Arousal	5 (1-7)	6 (2-7)	5 (2-8)	5 (1-8)	n.s.
Dominance	5 (3-8)	5 (3-9)	5 (1-6)	5 (2-8)	*(p=0.001)

Table S2: Mean Asynchrony descriptive statistics. Related to Figure 2 providing additional classical measures of coordination.

	same_synch	*same_asynch*	*distinct_synch*	*distinct_asynch*
Valid	***25***	***25***	***25***	***25***
Missing	***0***	***0***	***0***	***0***
Mean	***121.415***	***46.606***	***60.338***	***60.221***
Std. Deviation	***20.832***	***39.084***	***50.974***	***28.647***
Minimum	***80.910***	***-24.310***	***-111.840***	***-1.550***
Maximum	***152.940***	***113.490***	***131.490***	***114.860***

Table S3: Mean Asynchrony Repeated measures ANOVA. Related to Figure 2 providing additional classical measures of coordination.

Within Subjects Effects

Cases	Sum of Squares	df	Mean Square	F	p
Similarity	14078.297	1	14078.297	25.796	< .001
Residuals	13098.325	24	545.764		
Synchronicity	35087.284	1	35087.284	44.563	< .001
Residuals	18896.744	24	787.364		
Similarity ✻ Synchronicity	34868.093	1	34868.093	53.981	< .001
Residuals	15502.303	24	645.929		

Note. Type III Sum of Squares

Between Subjects Effects

Cases	Sum of Squares	df	Mean Square	F	p
Residuals	81635.615	24	3401.484		

Note. Type III Sum of Squares

Table S4: **Absolute Asynchrony *descriptive statistics.*** Related to Figure 2 providing additional classical measures of coordination.

Descriptive Statistics

	**same_synch**	**same_asynch**	**distinct_synch**	**distinct_asynch**
Valid	25	25	25	25
Missing	0	0	0	0
Mean	121.415	46.606	60.338	60.221
Std. Deviation	20.832	39.084	50.974	28.647
Minimum	80.910	-24.310	-111.840	-1.550
Maximum	152.940	113.490	131.490	114.860

Table S5: **Absolute Asynchrony *descriptive statistics.*** Related to Figure 2 providing additional classical measures of coordination.

Within Subjects Effects

**Cases**	**Sum of Squares**	**df**	**Mean Square**	**F**	**p**
Similarity	11992.002	1	11992.002	36.082	< .001
Residuals	7976.516	24	332.355		
Synchronicity	7605.933	1	7605.933	11.320	0.003
Residuals	16125.572	24	671.899		
Similarity ✻ Synchronicity	22312.592	1	22312.592	73.215	< .001
Residuals	7314.080	24	304.753		

Note. Type III Sum of Squares

Between Subjects Effects

Cases	Sum of Squares	df	Mean Square	F	p
Residuals	53231.097	24	2217.962		

Note. Type III Sum of Squares
